# Differences Between Women and Men in Phase I Cardiac Rehabilitation After Acute Myocardial Infarction: A Nationwide Population-Based Analysis

**DOI:** 10.1097/MD.0000000000002494

**Published:** 2016-01-22

**Authors:** Wen-Chih Lin, Chung-Han Ho, Li-Chen Tung, Chi-Che Ho, Willy Chou, Chun-Hou Wang

**Affiliations:** From the Department of Physical Medicine and Rehabilitation, Chi Mei Medical center, Chiali, Tainan, Taiwan (LW-C); Department of Medical Research, Chi Mei Medical Center, and Department of Hospital and Health Care Administration, Chia Nan University of Pharmacy and Science, Tainan, Taiwan (HC-H); Department of Physical Medicine and Rehabilitation, Chi Mei Medical Center, School of Medicine, Chung Shan Medical University, Taichung, Taiwan (TL-C); Department of Physical Medicine and Rehabilitation, Chi Mei Medical Center, Tainan, Taiwan (HC-C); Department of Physical Medicine and Rehabilitation, Chi Mei Medical Center, Chia Nan University of Pharmacy and Science, Tainan, Taiwan (CW); Department of Recreation and Health Care Management, Chia Nan University of Pharmacy and Science, Tainan, Taiwan (CW); and School of Physical Therapy, Chung Shan Medical University, and Physical Therapy Room, Chung Shan Medical University Hospital, Taichung, Taiwan (WC-H).

## Abstract

Although numerous studies have investigated gender-related differences in patients who have had an acute myocardial infarction (AMI), few studies have examined the gender-related differences among inpatients receiving Phase I inpatient cardiac rehabilitation following AMI.

Using data from the Taiwan National Health Insurance Research Database, this study analyzed 6713 adult patients who received inpatient cardiac rehabilitation following AMI between 2002 and 2011. The differences in comorbidity, medical service use, and prognosis between the male and female patients were analyzed to determine whether the comorbidities affecting their prognoses differed.

Female patients accounted for 23.18% of the sample, had a higher average age, and exhibited severe comorbidities; furthermore, they had significantly more days of hospitalization and days in an intensive care unit than did male patients. The gender-related differences in hospital mortality rate and 30-day mortality rate were nonsignificant, but female patients exhibited a significantly higher 1-year mortality rate. Moreover, the risk for 1-year mortality was higher among female patients with moderate or severe renal disease (odds ratio: 1.94, 95% confidence interval: 1.29–2.92) than among their male counterparts. However, the 1-year mortality rate for the female patients did not increase after all risk factors were adjusted.

Gender-related differences in age, comorbidity, and prognosis were confirmed in AMI patients receiving Phase I inpatient cardiac rehabilitation. In addition, gender-related differences were observed in the comorbidity risk factors affecting prognosis. However, being female did not affect the prognosis.

## INTRODUCTION

Acute myocardial infarction (AMI) is a common cardiovascular disease with a high mortality rate in both genders. More than 50% of older adult patients with AMI develop disabilities, leading to substantial consumption of medical resources.^1^ In addition, the occurrence of disabilities is particularly pronounced in women.^2^ Cardiac rehabilitation is implemented to prevent deconditioning among AMI patients due to being bedridden for prolonged periods.^3^ Cardiac rehabilitation, according to current developments, is a comprehensive, exercise-based approach proven to promote the recovery of daily functions and enhance patients’ quality of life.^4^ This type of rehabilitation is included in Class I treatment recommendation guidelines.^5,6^

Gender-related differences are a widely researched topic in the study of myocardial infarction. Biological differences between the genders lead to variations in pathogenesis,^7^ reactions to medication,^8^ clinical symptoms,^9^ and medical-resource utilization. Compared with male patients, female patients tend to receive fewer guideline-based treatments or evidence-based therapies^10^ and invasive procedures^11^; furthermore, female patients tend to exhibit a poorer prognosis.^12^

Gender is also a critical topic in the discussion of cardiac rehabilitation. Female patients receive less cardiac rehabilitation than male patients,^13^ with the difference reaching 36%.^14^ Female patients also exhibit a poorer prognosis, both physically and psychologically, than male patients after receiving cardiac rehabilitation.^15^ However, the targets in most of the related studies were patients receiving Phase II cardiac rehabilitation; the effect of gender among patients receiving Phase I cardiac rehabilitation has not been discussed in previous studies. The present study investigated differences in age distribution, comorbidities, medical-resource utilization, and prognosis among male and female patients receiving Phase I cardiac rehabilitation following myocardial infarction using data from a nationwide database. This investigation was conducted to determine whether a gender-related difference exists in prognostic comorbidity.

## METHODS

### Database

Taiwan's National Health Insurance Research Database (NHIRD), which includes data on approximately 99% of the population in Taiwan, was the data source for this study. The NHIRD, provided by the National Health Research Institute, includes patient information on disease diagnoses, procedures, surgical operations, rehabilitation, and prescriptions listed in health insurance claims. The diseases are coded according to the International Classification of Diseases, Ninth Revision, Clinical Modification (ICD-9-CM). In addition, doctors and medical institutions must follow the procedural codes for applying for health insurance reimbursement as established by the Bureau of National Health Insurance. In consideration of possible ethical violations related to the data, the privacy of each individual's information is protected using encrypted personal identification. Exemption was obtained from the institutional review board of Chi Mei Medical Center (IRB No. 10403-E06).

### Sample Population

The study subjects (age ≥ 18 years) were selected from the record of received inpatient cardiac rehabilitation following AMI from 2002 to 2011. The diagnosis of AMI was defined as a hospitalization with a primary or secondary discharge diagnosis code of ICD-9-CM 410.x. To avoid the possibility of the selection of study subjects who had not actually experienced AMI (eg, prior AMI patients who were admitted for a diagnostic or therapeutic intervention and were still coded for AMI), we excluded individuals whose period of hospitalization was less than 2 days. In Taiwan, inpatient cardiac rehabilitation involves consulting a physiatrist, who prescribes exercise training based on the patient's condition. Training ranges from passive joint exercises in bed to intensive exercise training in a movement-therapy facility. This study focused on exercise-based cardiac rehabilitation involving intensive exercise training under the guidance of a physical therapist and devices for detecting vital signs.

### Study Outcomes

The main event in this study was mortality, including in-hospital mortality, 30-day mortality, and 1-year mortality. The mortality was based on the death record of the inpatient claim dataset or the subject's final medical record plus withdrawal from the insurance program within 30 days without reenrollment.

### Risk Variables

In this study, the following risk variables were estimated: age at the index AMI admission, gender, Charlson comorbidities (excluding MI)^16^ within 1 year before the AMI diagnosis date, length of stay in the hospital (LOS), length of stay in an intensive care unit (ICU), and the use of AMI-related interventional procedures. The AMI-related interventional procedures were identified as ventilator support, intraaortic balloon pumping, extracorporeal membrane oxygenation, percutaneous coronary interventions, and coronary artery bypass graft (CABG).

### Statistical Analysis

Pearson Chi-square test or Fisher exact test was used to compare males and females in terms of categorical variables, such as age group, Charlson comorbidities index (CCI), AMI-related interventional procedures, and mortality. The mean age difference between males and females was analyzed by the Student *t* test. The LOS and the length of ICU stay of males and females were presented as the median with interquartile range and compared with the Wilcoxon ranked-sum test. The multivariable logistic regression model was applied to analyze the association between the Charlson comorbidities and 1-year mortality for overall and subgroup analysis. The odds ratios and 95% confidence intervals were calculated after adjusting for age, gender, and related variables. The significance level was set at *P* < 0.001 (2-tailed). All analyses were performed using the Statistical Analysis System (SAS) statistical software (version 9.4; SAS Institute, Inc., Cary, NC).

## RESULTS

From 2002 to 2011, a total of 6713 individuals (23.18%, women) were hospitalized due to AMI and received Phase I cardiac rehabilitation. The average age was 65.52 ± 12.71 years, and 53.63% of the patients were elderly adults. The average CCI was 1.31 ± 1.6. The top 3 comorbidities were diabetes (40%), congestive heart failure (25.44%), and cerebrovascular disease (9.74%).

Table 1 shows the difference in age and CCI according to sex. Female elderly adults accounted for a higher proportion of cases and had a higher average age (70 ± 10.9 years vs. 64.16 ± 12.9 years, *P* < 0.0001). Among these women, there were fewer cases of chronic lung disease, but significantly more cases of congestive heart failure, cerebrovascular disease, rheumatic disease, type 1 or type 2 diabetes, diabetes with chronic complications, and moderate–severe renal disease.

**TABLE 1:**
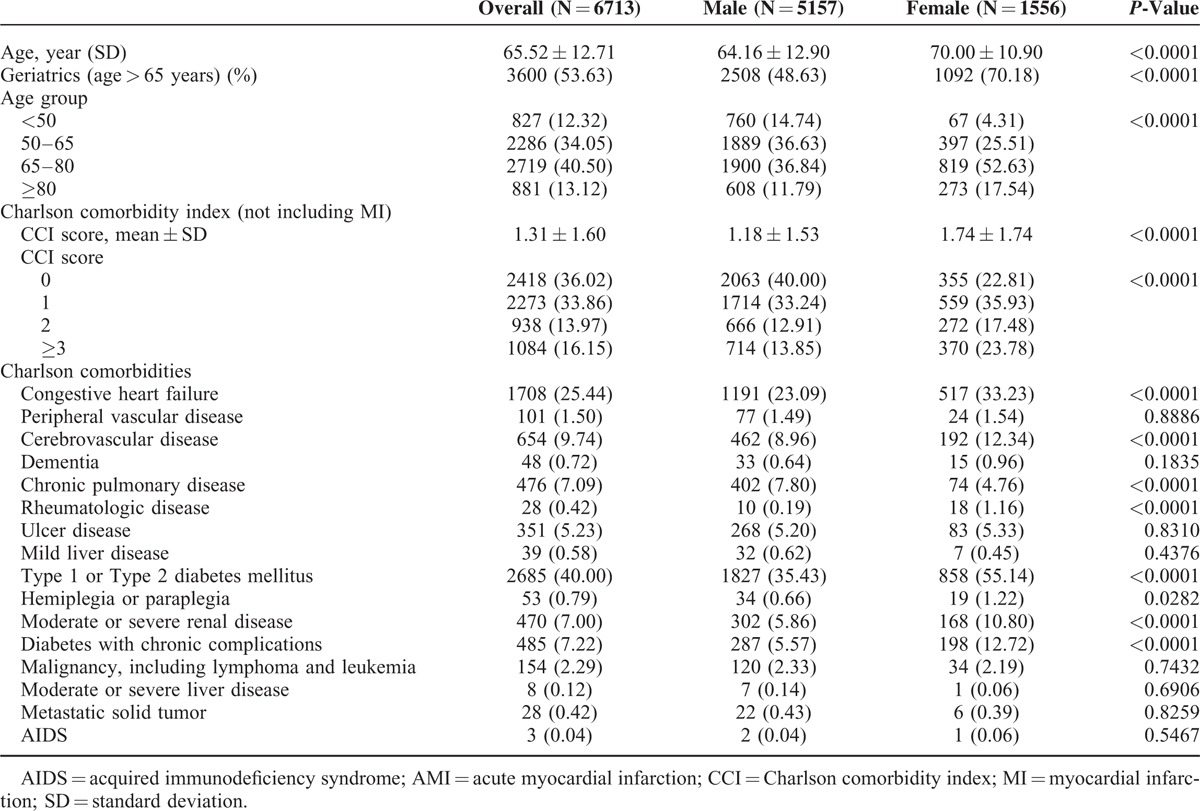
Gender-Related Differences in Clinical Characteristics of Patients Receiving Phase I Inpatient Cardiac Rehabilitation During Index AMI Hospitalization

As shown in Table 2, with regard to the medical treatments administered to patients receiving Phase I cardiac rehabilitation after an AMI, 52.57% of patients had coronary artery bypass grafting surgery, with no significant difference in the distribution between men and women. Percutaneous coronary intervention had been previously administered in 33.19% of cases, with more men than women receiving this treatment. With regard to the use of life-support systems, slightly more women required a respirator; more men required an intraaortic balloon pump and extracorporeal membrane oxygenation. Women had significantly more hospitalization days and significantly longer ICU stays. No significant differences between men and women were observed in the in-hospital mortality rate or 30-day mortality rate, although the 1-year mortality rate was significantly higher in women (18.51% vs. 14.16%, *P* < 0.0001).

**TABLE 2:**
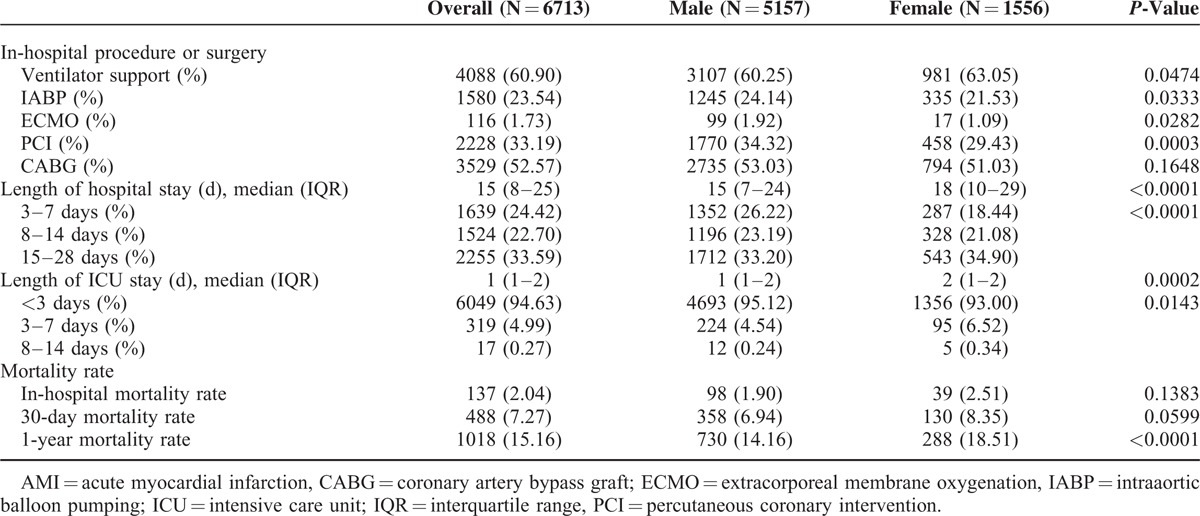
Medical Utilization and Outcomes of Gender-Related Difference in Patients Receiving Inpatient Cardiac Rehabilitation During Index AMI Hospitalization

For the patients receiving Phase I cardiac rehabilitation after an AMI, the 1-year mortality risk factors were congestive heart failure, peripheral vascular disease, cerebrovascular disease, moderate or severe renal disease, malignancy including lymphoma and leukemia, or metastatic solid tumor. Female patients with moderate–severe renal disease who received cardiac rehabilitation during hospitalization remained at high risk of 1-year mortality (odds ratio: 1.94, 95% confidence interval: 1.29–2.92, *P* < 0.05) (Table 3). When all factors were adjusted, being female did not increase the 1-year mortality rate, regardless of age (Table 4).

**TABLE 3:**
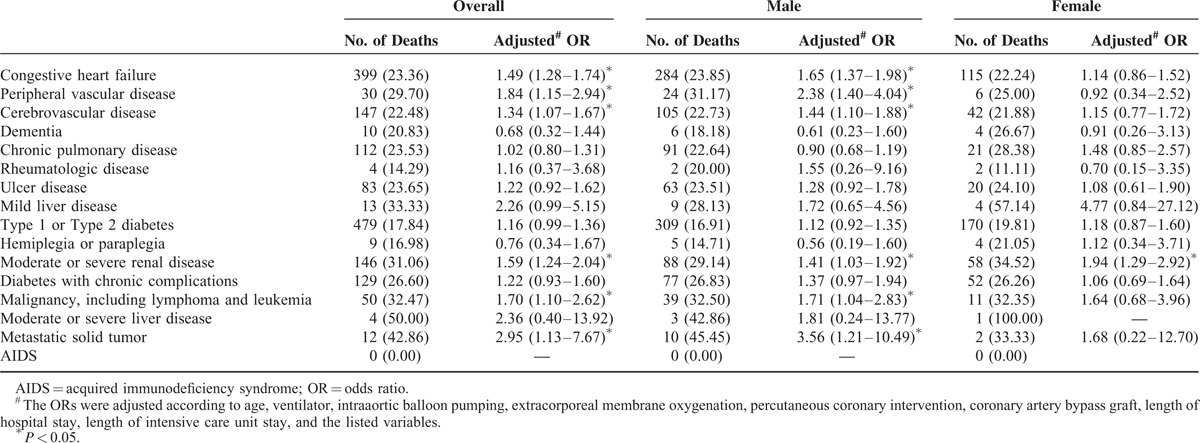
One-Year Mortality Among Charlson Comorbidities According to Gender

**TABLE 4:**
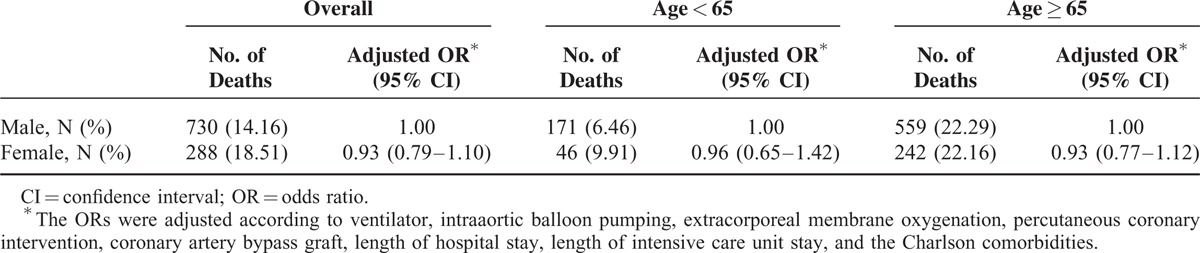
Adjusted OR for 1-Year Mortality, Female Versus Male

## DISCUSSION

This study analyzed data from a national database to determine the differences between male and female patients with AMI who received Phase I cardiac rehabilitation. The results indicated that fewer female patients received Phase I cardiac rehabilitation after AMI. In addition, female patients were generally older and developed more comorbidities. Fewer female patients received coronary angioplasty, and they had longer hospital stays. However, the in-hospital mortality and 30-day mortality rates did not differ between the sexes, although the female patients had a higher 1-year mortality rate. The comorbidities influencing the 1-year mortality rate differed between the sexes; specifically, moderate–severe kidney disease was a risk factor for 1-year mortality. However, being female was not a risk factor for 1-year mortality.

In the present study, patients who had an AMI were at higher risk for various cardiovascular comorbidities, such as diabetes, heart failure, and stroke,^17^ and noncardiac comorbidities, such as chronic obstructive pulmonary disease.^18^ These results are consistent with findings reported in the literature. Because the prevalence of various comorbidity risk factors for mortality in patients who have had an AMI differs between the sexes,^19^ the level of influence that such comorbidities have on the mortality rate also differs according to sex. In the present study, the 1-year mortality rate was higher for AMI patients who also had kidney disease. This observation was particularly evident in female AMI patients. This could be attributed to women typically being at higher risk for contrast-induced nephropathy. Thus, the presence of kidney disease among the female patients may have affected their prognosis considerably. However, further investigation is required to determine whether the influence of moderate or severe renal disease on the mortality rate of AMI patients differs between the sexes and whether it is more pronounced in women than in men after Phase I inpatient rehabilitation.

Patients who have had an AMI typically have other comorbidities, which usually influence patient prognosis.^18^ Although the CCI was not developed to assess the risk of patients with AMI,^20^ this index can still be used to predict the in-hospital and 1-year mortality rates of patients with AMI, in which the risk for in-hospital mortality correlates positively with the CCI.^21^ In the present study, the CCI was higher for women than for men, although the in-hospital mortality rate did not differ significantly between the sexes. Whether this result was influenced by Phase I cardiac rehabilitation requires further investigation. All patients examined in the present study had received Phase I cardiac rehabilitation following hospitalization for AMI, which differs from the findings of other relevant studies.

Previous studies have indicated that the aerobic capacity of women receiving Phase I cardiac rehabilitation is relatively lower than that of men.^22^ In addition, as compared with their male counterparts, women receiving Phase I cardiac rehabilitation are typically older, have a lower socioeconomic status, and have a poorer prognosis.^15^ However, the factors contributing to referral to Phase I cardiac rehabilitation are typically more evident in women than in men.^14^ Therefore, understanding the characteristics of female patients receiving Phase I cardiac rehabilitation and the influences that such characteristics have on their prognosis can contribute to the development and implementation of effective secondary prevention measures.

This study had the following limitations. First, because the research data were obtained from Taiwan's NHIRD, information on patients not enrolled in Taiwan's National Health Insurance program was not accessible. Furthermore, information on numerous pathogenic and prognostic factors, such as body mass index, exercise habits, smoking and drinking habits, family history, and socioeconomic status, could not be obtained. In addition, only those patients with a primary diagnosis code of 410.xx were analyzed. However, the arrangement of primary and secondary codes was subjectively biased, and thus the possibility of coding errors was unavoidable, which may have influenced the results. In addition, the type of AMI (ST-segment elevation myocardial infarction or non-ST-segment elevation myocardial infarction), the number of infarcted blood vessels, and the extent of infarction could not be determined from the primary diagnosis code alone. And the information on the patients’ prescription frequency, intensity, type of training activity, and time of cardiac rehabilitation intervention was unknown. Finally, the targets of analysis in the present study were short-term outcomes (ie, in-hospital mortality, 30-day mortality, and 1-year mortality rates). Consequently, the analysis did not account for whether patients underwent cardiac rehabilitation following discharge; nor did it account for the influence of long-term outcomes.

## CONCLUSION

This study conducted a national database analysis to determine the gender-related differences among patients receiving inpatient Phase I cardiac rehabilitation following an AMI. The results indicated that fewer female patients with AMI received Phase I cardiac rehabilitation. In addition, female patients were generally older, developed more complications, and exhibited a higher 1-year mortality rate. In particular, the 1-year mortality rate of female patients with both AMI and mild–severe kidney disease was higher than that of female patients without kidney disease. Physicians should be more active in encouraging this group of patients to undergo Phase I outpatient cardiac rehabilitation and provide them with secondary prevention strategies to reduce their risk of mortality. Furthermore, medical teams must pay particular attention to the differences between male and female patients with AMI during their in-hospital acute phase of rehabilitation to optimize their treatment plans and prognostic assessment.
